# Preparing for the Unexpected, Supporting the Vulnerable—A Perspective From Lebanon and Sri Lanka

**DOI:** 10.1016/j.ekir.2023.01.022

**Published:** 2023-01-31

**Authors:** Sabine Karam, Vindya N. Gunasekara, Pauline Abou Jaoudeh, Eranga Wijewickrama

**Affiliations:** 1 Division of Nephrology and Hypertension, University of Minnesota, Minneapolis, Minnesota, USA; 2 Lady Ridgeway Hospital For Children, Colombo, Sri Lanka; 3 Division of Pediatric Nephrology, Lebanese American University, Beirut, Lebanon; 4 Faculty of Medicine, University of Colombo, Colombo, Sri Lanka; 5 National Hospital of Sri Lanka, Colombo, Sri Lanka

Chronic kidney disease (CKD) is a major global public health problem and is projected to become the fifth highest contributor to years of life lost by 2040.^1^ Morbidity and mortality of patients with CKD are profoundly impacted by social determinants of health,^2^ and among all noncommunicable diseases, the prognosis of patients with CKD might be the most susceptible to socioeconomic status, political contexts, environmental factors, and their stability. Recently, many disruptions have impacted global access to kidney diagnostic modalities, treatments, and care along with devastating effects on human health in general and wellbeing. These disruptions include, but are not limited to, the ongoing COVID-19 pandemic, armed conflicts, economic catastrophes as are the cases in Lebanon and Sri Lanka, and natural and man-made disasters such as the Beirut blast in Lebanon. In addition, changing climate patterns with extreme weather events have been linked to increased risk of both the development and exacerbation of CKD.^3^ Consequently, startling inequities in kidney care, already established with significant gaps in oversight, funding, and infrastructure to support care for patients with kidney disease, are likely to deepen further, especially in low-income countries and low-middle-income countries (LMICs).^4^ Therefore, the theme of this year’s World Kidney Day “Kidney Health for All-Preparing for the Unexpected, Supporting the Vulnerable!,” is indeed, and without a doubt, timely and suitable.

### Current Threats Affecting the Development and Sustainability of Kidney Health

#### Impact of the COVID-19 Pandemic

The COVID-19 pandemic represents the most unexpected and impactful threat that affected global health in recent times. It is difficult to find any patient group that was not affected by the pandemic and patients with CKD were no exception. Among patients with CKD, those receiving kidney replacement therapy (KRT) and those who had received kidney transplants constitute the most fragile groups at risk of acquiring and developing complications of the infection. During the initial phase when there were countrywide lockdowns, many individuals were completely deprived of accessing basic and specialized kidney care, including KRT. Furthermore, the demand for KRT increased because of the increased incidence of acute kidney injury and the rapid progression of existing CKD because of the COVID-19 infection. It was the health systems of the low-income countries and the LMICs, which were already overwhelmed by the increased demand for KRT, that were the most affected by the pandemic, resulting in the widening of the already existing global inequities in the availability of KRT. A global online survey of hemodialysis (HD) units conducted during the initial phases of the pandemic by the International Society of Nephrology and the Dialysis Outcomes and the Practice Patterns Study reported a significantly higher percentage of missed HD treatment in low-income countries (64%) and LMICs (67%) compared with the upper middle-income countries (31%) and high-income countries (6%).^5^ It also revealed limited access to HD, intensive care unit care and mechanical ventilation among hospitalized patients on chronic dialysis with COVID-19, particularly in low-income countries and LMICs as compared with upper middle-income countries and high-income countries.

#### Conflicts and Population Displacement

Wars and armed conflicts, like any other adversities, have devastating effects on the sick by interfering with access to life-saving therapies and medications. Syria, Afghanistan, Yemen, Ethiopia, South Sudan, and Myanmar are some of the countries affected by ongoing civil wars and civil unrest. On February 24, 2022, Russia invaded Ukraine, taking the war to the heart of Europe for the first time since World War II, which ended in 1945. As in other situations, the ongoing war in Ukraine has resulted in massive destruction of the infrastructure of the country leading to mass displacement of the population to neighboring countries. Kidney patients are a vulnerable group greatly affected by wars because their treatment depends heavily on the presence of sophisticated technology and well-trained personnel. Services such as HD require a continuous supply of water, electricity, and consumables, which are frequently disrupted in a conflict situation. Often, there are difficulties for patients in reaching the dialysis units because of safety concerns and disruptions in the transport facilities. Even the continuation of home-based therapies such as peritoneal dialysis is not possible without access to clean water, adequate lighting, and a continuous provision of necessary materials. Patients frequently choose to move out from areas of conflict because of the disruptions to their treatments, but there is no guarantee that things will be better in the new environment. They need to travel long distances studded with multiple dangers because they would be cut off from their usual treatments and follow-up for days and need to find support in unfamiliar surroundings with language and cultural barriers.^6^^,^^7^ In addition, in most countries, refugees and undocumented immigrants are excluded from government policies regarding KRT coverage and resort to emergency-HD only,^8^ with subsequent higher morbidity, mortality, and costs.^9^

#### Economic Crises

In the past year, the World Bank has warned about a rapid deterioration of growth prospects, coupled with rising inflation and tightening financing conditions, leading to an impending global economic crisis and recession.S1 The examples of Sri Lanka and Lebanon are deeply revealing of what many parts of the world could expect if these predictions were to hold true. Sri Lanka’s economy was already on a downward slide before the COVID-19 pandemic. With the foreign earnings from tourism and overseas workers coming to a standstill, the official reserves dropped from US$7.6 billion in 2019 to less than US $400 million in June 2022 and Sri Lanka announced external debt suspension in April 2022.S2 The severe shortage of foreign currency led to severe shortages of fuel, cooking gas, and medicines, among other essential items. Schools were closed, and workers were requested to work from home because of the widespread disruptions in private and public transport. Patients with advanced CKD and kidney transplants were the 2 main groups that were seriously affected by the financial crisis. There were severe shortages in immunosuppressive medications such as tacrolimus, cyclosporine, and mycophenolate used by kidney transplant patients. Most kidney transplant recipients in Sri Lanka depend on the state sector hospitals for these medications, where these are provided free of charge. Many could not afford to buy them from the private sector and had to discontinue them, resulting in catastrophic results. There were disruptions in the supply of consumables for HD and peritoneal dialysis with many units reducing the dialysis frequency and the number of peritoneal dialysis exchanges in patients. There were prolonged interruptions of electricity, at some points extending up to 8 to 10 hours per day, further affecting the functioning of the HD units. Even when the dialysis units were functioning, reaching the dialysis units was extremely challenging because of the lack of public transport resulting from fuel shortages. There was some relief though through the purchase of essential medicines made through the credit lines provided by the Indian government and the Asian Development Bank. Furthermore, in most hospitals, the services were maintained with the help of private donations made in response to personal requests of the health care staff.

Similarly, since 2019, Lebanon has been experiencing a severe economic and financial crisis that has been described in a report by the World Bank as one of the worst the world has seen in more than 150 years. The gross domestic product per capita dropped by 36.5% between 2019 and 2021, and Lebanon was reclassified in July 2022 by the World Bank as a LMIC, down from upper middle-income country status.S3 This has resulted in a major devaluation of the national currency vis-à-vis the US dollar exceeding 95%, and it has not been without dire consequences for the health care sector, and most notably, the nephrology community. Before the economic crisis, Lebanon boasted of one of the highest densities of physicians, and most notably nephrologists, per capita in the world at 190 for a population of 6 million or 32 per million population. Comparatively, the density is 24.4 per million population in Western Europe and 18.1 per million population in North America.S4 Unfortunately, since the beginning of the crisis, it has been estimated that approximately 70 adult nephrologists and 2 out of 8 pediatric nephrologists have left the country permanently. Barely half of the remaining workforce continues to practice because their main source of income is the Lebanese Ministry of Health reimbursement for dialysis, which usually occurs in local currency and has become insufficient in value to cover their basic life needs. Moreover, Lebanon was a pioneer in terms of universal coverage for hemodialysis because it was implemented in the country since the early 1970s with peritoneal dialysis added around 1996. However, the crash in the value of the local pound and the extreme inflation have undercut the country’s ability to pay for importing medical supplies essential for the provisioning of KRT and have made the government’s reimbursement modality in local currency insufficient to cover the cost. Consequently, difficult compromises had to be made by dialysis units to cope with the emerging strenuous situation. These compromises include implementing stringent quotas regarding the number of patients who can be treated in-center, reducing the frequency of dialysis sessions, and even charging the patients with significant additional out-of-pocket expenditures, in a country where the economic crisis has plunged more than 50% of the population under poverty level with 78% of one of the most vulnerable groups, the older persons living in multidimensional poverty.S5 Similar to Sri Lanka, the severe shortage of foreign currency in addition to severe restrictive monetary policies with the lifting of medical subsidies by the Central Bank, have dramatically affected the ability to access proper medical care for all, in terms of hospital bills, imported medical supplies, and provision of medications in addition to severe shortages of fuel and cooking gas among other essential items. This unfortunate situation in Lebanon is a reminder of the fragility and in many countries even the lack of support systems for dialysis. Indeed, globally, public funding for KRT with no fees at the point of delivery was available in 2018 in 43% of countries only,^4^ and it is estimated that only between 27% and 53% of the population who needs KRT receive it.S6 The progression of poverty in this country and others is also concerning for a worsening of CKD prevalence and outcomes.S7 The dual economic impact of the COVID-19 pandemic and the massive Port of Beirut explosion in August 2020 have amplified the crisis further. On August 4, 2020, one of the largest non-nuclear explosions in history pulverized the port and damaged over half the city of Beirut, Lebanon, causing at least 218 deaths, 7000 injuries, and leaving an estimated 300,000 people homeless.S8 Three hospitals in the vicinity of the blast were destroyed and 2 of them were partially damaged.S9 Among the hospitals heavily impacted, Saint George Hospital University Medical Center, is one of the largest healthcare facilities in the country, one of the few tertiary care centers and a major teaching institution. It also hosts one of the largest dialysis units and 1 of only 2 pediatric dialysis units in Lebanon. The hospital was so severely damaged that it had to cease its activities completely for more than a month, a first in its 150 years of existence, depriving a significant part of the city of its services. Dialysis patients had to be urgently evacuated and relocated to other units, thereby disrupting their usual functioning. Moreover, an alarming percentage of these patients displayed significant moral distress several months after the blast.S10 Finally, nondialysis patients with CKD and kidney transplant recipients were also adversely affected in the aftermath because the pre-existing medications shortage considerably worsened with the destruction of the government’s main drug warehouse in Beirut, Lebanon.

#### Natural Disasters and Climate Change

Not only man-made disasters such as the Beirut explosion, but also natural disasters and extreme weather events, which are on the rise can have negative repercussions on the incidence, progression, and provision of care to patients with CKD. When Hurricane Katrina occurred, 44% of patients living in the affected area reported missing at least 1 dialysis session and almost 17% reported missing 3 or more dialysis sessions.S11 In addition, there was a 21% decline in kidney transplant services in the area a year later.S12 A review of outcomes of patients registered in the United States Renal Data system as requiring maintenance dialysis over a period of 10 years in 1 of 108 hurricane-afflicted counties revealed a higher mortality risk in the 30 days after a hurricane.S13 The worldwide increase in temperature has resulted in a marked increase in heat waves with a markedly increased risk for morbidity and mortality and notably at the kidney site.S14 Finally, pollution is another significant determinant of kidney health, with exposure to particles with a mass median of aerodynamic diameter <2.5 μm, associated with increased incidence of CKD, higher level of albuminuria, progression of CKD, and kidney failure.S15,S16

#### Concerns Related to Special Populations

Disasters and economic downfalls of countries affect all segments of a community, but some groups like children, the elderly, and the mentally challenged people are especially susceptible. Substandard medical support can be particularly deleterious to the growth development, psychosocial wellbeing, and mental health of children with its long-term effect yet to be defined. In addition, the provision of services to children can be more severely influenced because of unique age-related issues, dependency on a caregiver, and scarcity of specialized pediatric health care expertise.S17 More than adult nephrology, the pediatric workforce suffers from severe shortages even in high-income settings.S18,S19 During times of disaster, the requirement of a parent or a guardian to gather information and deliver medical care creates major challenges for unaccompanied children who get separated from their families. For such children, the inability to protect themselves and to express their needs not only paves way for exploitation and further psychological and physical traumas but also delays access to a specific treatment. In addition, most of the small-size pediatric vascular catheters and dialysis consumables do not generate adequate profit in business and therefore were not freely available in both Lebanon and Sri Lanka even before their crises. Therefore, providing dialysis to infants and young children becomes extremely challenging as the supply chains get progressively compromised. In addition, the needs of the relatively much smaller pediatric population can be easily ignored, when operating with restricted budgets.

### Opportunities to Prepare for the Unexpected and Support the Vulnerable

This unfortunate series of events should serve as a catalyst to adopt a series of measures to try to curb the increase in CKD prevalence, ensure the sustainability of essential and life-saving kidney care modalities, protect the vulnerable, and prepare for the unexpected. In this setting, the requirements of the most vulnerable groups such as pediatric patients need prioritization from the onset of any care planning. In the case of armed conflicts, there should be reinforcement and revision of the Geneva Convention Treaties to protect the sick and wounded with a specific clause pertaining to patients receiving KRT along with the establishment of support funds for displaced populations in need of kidney care. In addition, considering the increased number of illegal immigrants and refugees, there should be establishment of government policies to provide unrestricted kidney care to them. Moreover, there should be design and implementation of disaster preparedness plans globally. Finally, the reinforcement of conventions and treaties to address global climate change is also necessary (see Figure 1).

**Figure 1:**
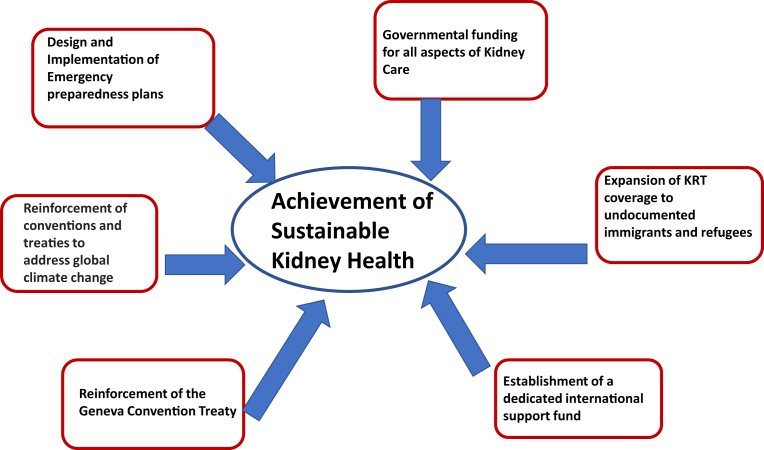
The reinforcement of conventions and treaties to address global climate change. KRT, kidney replacement therapy.

### Conclusion

The numerous threats faced by the global kidney community present a unique opportunity to identify solutions to existing and impending challenges in the care and delivery of health care to vulnerable people living with or at risk of kidney diseases. Building capacities in human resources, sustainable policies, and strategic partnerships will be necessary to protect any existing gains in the war against kidney diseases, prevent any threats, and ultimately eradicate its global burden.

## Disclosure

All the authors declared no competing interests.

## Supplementary Material

Spplementary File (PDF)

**Supplementary References.**
