# Supplementary material for: Preparing for the Unexpected, Supporting the Vulnerable—A Perspective From Lebanon and Sri Lanka
Source: Kidney Int Rep. 2023 Jan 31;8(3):383–7. doi: 10.1016/j.ekir.2023.01.022 (PMC10014372; doi:10.1016/j.ekir.2023.01.022)
Supplement: Spplementary File (PDF) [file mmc1.pdf]

Additional References:

S1.https://openknowledge.worldbank.org/bitstream/handle/10986/38019/Global-Recession.pdf.Last accessed on January 17th 2023.

S2. https://www.worldbank.org/en/country/srilanka/overview. Last accessed on January 17th 2023.

S3. https://www.worldbank.org/en/country/lebanon/overview.

S4. Riaz P, Caskey F, McIsaac M, et al. Workforce capacity for the care of patients with kidney failure across world countries and regions. BMJ Glob Health. 2021 Jan;6(1):e004014.

S5. https://www.unescwa.org/sites/default/files/news/docs/21-00634-_multidimentional_poverty_in_lebanon_-policy_brief_-_en.pdf. Last accessed on January 17th 2023.

S6. Bharati J, Jha V, Levin A. The Global Kidney Health Atlas: Burden and Opportunities to Improve Kidney Health Worldwide. Ann Nutr Metab. 2020;76(Suppl. 1):25–30.

S7. Norris KC, Beech BM. Social Determinants of Kidney Health: Focus on Poverty. CJASN. 2021 May 8;16(5):809–11.

S8. "They Killed Us from the Inside" An Investigation into the August 4 Beirut Blast (PDF) (Report). Human Rights Watch. 2021. p. 706. ISBN 978-1-62313-931-5.

S9. "Lebanon eyes state of emergency after deadly Beirut blast: Live". Al Jazeera English. 5 August 2020. Archived from the original on 5 August 2020. Last accessed January 17th 2023.

S10. Karam S, Ghantous Z, Ibrahim R, et al. POS-738 ASSESSMENT OF DEPRESSION, ANXIETY, SUICIDALITY AND POST-TRAUMATIC STRESS DISORDER IN LEBANESE PATIENTS ON HEMODIALYSIS FOLLOWING THE BEIRUT EXPLOSION. Kidney International Reports. 2022 Feb;7(2):S318.

S11. Anderson AH, Cohen AJ, Kutner NG, et al. Missed dialysis sessions and hospitalization in hemodialysis patients after Hurricane Katrina. Kidney International. 2009 Jun;75(11):1202–8.

S12. Kopp JB, Ball LK, Cohen A, et al. Kidney Patient Care in Disasters: Lessons from the Hurricanes and Earthquake of 2005. CJASN. 2007 Jul;2(4):814–24.

S13. Blum MF, Feng Y, Anderson GB, et al. Hurricanes and Mortality among Patients Receiving Dialysis. JASN. 2022 Sep;33(9):1757–66.

S14. Johnson RJ, Sánchez-Lozada LG, Newman LS, et al. Climate Change and the Kidney. Ann Nutr Metab. 2019;74(Suppl. 3):38–44.

S15. Bowe B, Xie Y, Li T, et al. Particulate Matter Air Pollution and the Risk of Incident CKD and Progression to ESRD. J Am Soc Nephrol. 2018 Jan;29(1):218–30.

S16. Blum MF, Surapaneni A, Stewart JD, et al. Particulate Matter and Albuminuria, Glomerular Filtration Rate, and Incident CKD. CJASN. 2020 Mar 6;15(3):311–9.

S17. Sever L, Pehlivan G, Canpolat N, et al. Management of pediatric dialysis and kidney transplant patients after natural or man-made disasters. Pediatr Nephrol. 2023 Feb;38(2):315–25.

S18. Glenn D, Ocegueda S, Nazareth M, et al. The global pediatric nephrology workforce: a survey of the International Pediatric Nephrology Association. BMC Nephrol. 2016 Dec;17(1):83.

S19. Ashoor I, Weidemann D, Elenberg E, et al. The Pediatric Nephrology Workforce Crisis: A Call to Action. The Journal of Pediatrics. 2021 Dec;239:5-10.e4.
